# Tissue-specific accumulation of Na^+^ in leaves of 2 tomato species is linked to differences in symplastic transport

**DOI:** 10.1093/plphys/kiag779

**Published:** 2026-11-04

**Authors:** Lidor Shaar-Moshe, Daniel E Runcie, Siobhán M Brady

**Affiliations:** Department of Plant Biology and Genome Center, University of California, Davis, One Shields Avenue, Davis, CA 95616, United States; Department of Evolutionary and Environmental Biology, Faculty of Natural Sciences, Institute of Evolution, University of Haifa, 199 Abba Khoushy Avenue, Mount Carmel, Haifa 3498838, Israel; Department of Plant Sciences, University of California, Davis, One Shields Avenue, Davis, CA 95616, United States; Department of Plant Biology and Genome Center, University of California, Davis, One Shields Avenue, Davis, CA 95616, United States; Howard Hughes Medical Institute, UC Davis, 451 Health Sciences Dr., Davis, CA 95616, United States

## Abstract

Soil salinization is a growing global threat that limits crop productivity. To cope with sodium (Na^+^) stress, plants have evolved tolerance mechanisms, including excluding Na^+^ from shoot tissues and tolerating elevated Na^+^ within shoots through tissue- and cellular-level mechanisms. Most current knowledge of Na^+^ accumulation comes from organ- or whole-plant measurements that lack the spatial resolution needed to resolve cellular tolerance mechanisms. Here, we used histological approaches to map leaf Na^+^ distribution in tomato (*Solanum*) species with contrasting salt-tolerance strategies. In the moderately salt-tolerant, Na^+^-excluding domesticated tomato (cv. M82), Na^+^ was largely confined to the bundle sheath, whereas in salt-tolerant, Na^+^-including wild relatives, Na^+^ accumulated throughout the blade mesophyll. Consistent with these cell population-specific Na^+^ patterns, M82, but not *Solanum pennellii*, exhibited reduced symplastic transport and plasmodesma permeability under salt stress. A genetic screen combined with transcriptome profiling implicated *Plasmodesmata-Located Protein 1* (*PDLP1*), a regulator of callose-mediated plasmodesma closure, in establishing symplastic domains in M82 that restrict Na^+^ movement into the mesophyll. Moreover, *PDLP1* expression was negatively correlated with mesophyll Na^+^ levels across wild and domesticated tomatoes. Collectively, these results link cellular Na^+^ enrichment patterns to symplastic connectivity and suggest that *PDLP1*-mediated regulation of plasmodesmata contributes to leaf-level salt-tolerance strategies.

## Introduction

Salt stress is a growing environmental constraint on crop productivity, particularly in coastal, semi-arid, and arid regions. Climate change exacerbates soil salinization, which limits plant growth, especially in new shoots, by reducing the plant's capacity to take up water. Additionally, excessive accumulation of sodium ions (Na^+^) within plants is toxic to key biochemical processes and accelerates the senescence of mature leaves (Munns and Tester 2008; van Zelm et al. 2020). Approximately 33% of irrigated agricultural land is already salt-affected, and this area is expanding by ∼1–2 million hectares per year, in part due to the increasing use of treated wastewater and marginal-quality water for irrigation (Jenks et al. 2007; Hopmans et al. 2021). Thus, understanding and optimizing the molecular and cellular mechanisms underlying salt stress tolerance is critical for breeding crops with sustained productivity on salt-affected agricultural lands.

Upon entering the root epidermis through nonselective cation channels (Demidchik and Maathuis 2007), Na^+^ can move into inner root cell layers via plasmodesmata (symplastic transport), through extracellular cell wall spaces (apoplastic transport), or by repeated membrane crossing from cell to cell mediated by ion channels and transporters (transcellular transport) (van Zelm et al. 2020). To maintain cellular function and prevent damage to membranes and proteins (Benito et al. 2014), plants regulate cytosolic Na^+^ levels by exporting Na^+^ from root cells or sequestering it in root cell vacuoles through transporters and antiporters that sustain Na^+^/K^+^ balance (Almeida et al. 2017). Recently, Ramakrishna et al. (2025) demonstrated that the highly selective sodium/proton antiporter SALT OVERLY SENSITIVE 1 localizes not only to the plasma membrane but also to late endosomal/prevacuolar and vacuolar membranes, supporting a direct role in intracellular Na^+^ compartmentation in addition to plasma-membrane Na^+^ extrusion. After entering the root stele, Na^+^ is loaded from stelar/xylem parenchyma cells into the xylem, from which it is transported with the transpiration stream through the stem and petiole, and may accumulate in the leaf blade over time. Since only a small portion of Na^+^ is recycled from shoot to root via the phloem, most Na^+^ reaching the shoot remains there, making the leaf blade the main site of Na^+^ toxicity for most plants (Munns and Tester 2008). Na^+^ that accumulates in the blade mesophyll must first cross the bundle sheath, a parenchymatous cell layer surrounding the leaf's vascular bundles, either symplastically or apoplastically. In some grass species, the bundle sheath cell walls are suberized and have been shown to restrict the movement of apoplastic dyes (Botha et al. 1982). However, another study using different apoplastic dyes challenged this finding (Mertz and Brutnell 2014), raising questions about the role of the bundle sheath as an apoplastic barrier.

Despite the well-established negative effects of excessive Na^+^ accumulation on photosynthesis, surprisingly little is known about the specific cellular domains in which Na^+^ accumulates within leaves. A possible explanation for this knowledge gap is that salt-stress studies have largely overlooked the functional heterogeneity of distinct cell populations that comprise the above-ground plant body. Additionally, analyses of Na^+^ levels are traditionally conducted on bulk tissues using destructive (Hansen et al. 2013) or indirect methods (Arino-Estrada et al. 2019; Perelman et al. 2020), which either eliminate our ability or lack adequate resolution to identify differential Na^+^ accumulation in distinct cell populations and the underlying genetic mechanism(s). Thus, bridging these knowledge gaps requires elucidating the functions of distinct cell populations, the cellular patterning of Na^+^ accumulation, and the integration of these processes across the cellular and tissue levels to facilitate whole-plant salt tolerance.

Tomato is among the world's most produced vegetable crops (Ritchie et al. 2023), cultivated in many arid and semi-arid regions, where brackish or saline water is used for irrigation. Under such irrigation, tomato growth and yield decline as water salinity increases (Zhai et al. 2015). Yet, their wild relatives are native to arid regions with high soil salinization and thus adapted to better withstand these unfavorable growth conditions (eg, *S. pimpinellifolium*, *S. pennellii*, *S. peruvianum*) (Bonarota et al. 2022). It has long been known that at the organ level, the domesticated tomato species attempts to exclude salt from its leaves, while wild salt-tolerant tomatoes, including *S. pennellii*, accumulate salt in their leaves (Tal and Shannon 1983). To dissect the phenotypic diversity and identify genetic loci regulating salt stress tolerance, researchers have utilized an introgression line (IL) population generated by crossing *S. pennellii* LA0716 with the processing inbred cv. M82 (Eshed and Zamir 1994, 1995). This publicly available population has been genotyped at ultrahigh density to determine the exact gene content harbored within each line (Chitwood et al. 2013). A wide range of biologically and agronomically relevant traits, including salt stress tolerance (Frary et al. 2010; Frary et al. 2011; Li et al. 2011; Uozumi et al. 2012), have been previously analyzed using this IL population. However, the cellular and genetic basis for the divergent salt tolerance strategies of *S. pennellii* and *S. lycopersicum* cv. M82 (hereafter referred to as M82) is poorly understood.

In this study, we sought to investigate whether the divergent Na^+^ accumulation observed at the organ level between the 2 tomato species is also evident at the level of cellular spatial domains. To achieve this, we employed histological, genetic, and transcriptomic approaches to investigate the cellular domains of Na^+^ accumulation in tomato leaves and the mechanisms underlying their regulation. Our findings suggest that divergent patterns of foliar Na^+^ accumulation across tomato species are associated with differences in symplastic transport and *PDLP1* expression, thereby providing a mechanistic model for how cellular Na^+^ accumulation domains are spatially regulated in leaves.

## Results

### M82 and *S. pennellii* exhibit divergent Na^+^ accumulation patterns in their leaves

*S. lycopersicum* cultivars are generally moderately tolerant to salt stress, show reduced growth under such conditions, and use a Na^+^ exclusion mechanism to prevent Na^+^ buildup in their shoots. In contrast, their salt-tolerant wild relatives, including *S. pennellii,* exhibit less growth reduction under salt stress and typically employ a Na^+^ inclusion mechanism in their shoots (Dehan and Tal 1978; Shannon et al. 1987). We first tested whether the 2 tomato species display different salt tolerance strategies (ie, excluder vs. includer) under our experimental conditions. We exposed the plants to salt stress, starting at the early vegetative stage with 3 to 4 fully expanded leaves, and gradually increased the salt concentration in the nutrient-rich water by irrigating daily with 25, 50, and 75 mM NaCl until reaching a target concentration of 100 mM NaCl. This concentration was maintained for 9 d, during which plants developed 7 to 9 leaves. Overall, the 5-week-old plants were exposed to salt stress for 2 wks. During the salt treatment, control plants were irrigated daily with nutrient-rich water (electrical conductivity of 1.4 dS/cm) and developed the same number of leaves as the treated plants. Consistent with previous reports, after 2 wks of salt stress conditions, M82 showed a significant decrease in total biomass and leaf relative water content, along with an increase in osmotic adjustment (OA) (Figure S1). This adaptation was likely achieved through the synthesis of compatible solutes, as K^+^ levels decreased moderately and Na^+^ levels remained relatively low compared with *S. pennellii* (Figure S2a and b). In contrast to M82, *S. pennellii* maintained its whole-plant biomass under salt stress and showed a minor increase in OA (Figure S1), likely due to the high Na^+^ levels accumulated in its shoots, as K^+^ levels sharply decreased under salt stress (Figure S2a and b). Together, these physiological (whole-plant and whole-leaf) and whole-leaf bulk ion measurements indicate that, under our experimental conditions, M82 exhibited an excluder-like shoot response, restricting Na^+^ accumulation in leaves while largely maintaining leaf K^+^. In contrast, *S. pennellii* exhibited an includer-like response, accumulating high leaf Na^+^ and tolerating a lower K^+^/Na^+^ ratio under salt stress (Figure S2c and C’), consistent with previous reports for other tomato cultivars and wild relatives (Tal and Shannon 1983; Tahal et al. 2000; Albaladejo et al. 2017).

We hypothesized that these divergent salt tolerance strategies result from variations in cellular regulation of Na^+^ transport and accumulation between the 2 Solanum species. To test the hypothesis that cellular Na^+^ accumulation differs between species, we used CoroNa Green, a Na^+^-specific fluorescent dye (Shao et al. 2020). Because CoroNa Green fluorescence and membrane impermeability depend on living, intact cells, it was essential to ensure that cells in the sections remain viable during staining and imaging (Figure S3a and b). Using this Na^+^-specific dye, we found that, in M82 leaves exposed to 2 wks of salt stress, Na^+^ predominantly accumulated in the bundle sheath, identified by the presence of amyloplasts, and in adjacent parenchyma cells on the abaxial side of the leaf (Fig. 1a, Figure S3c-f). Moreover, this pattern supports growing evidence for differential ion accumulation and cell population-specific gene expression and function, implicating the bundle sheath as a regulatory, selective barrier that can restrict ion transport (Shapira et al. 2009; Aubry et al. 2014; Wigoda et al. 2014). In contrast, in *S. pennellii*, Na^+^ mainly accumulated in the mesophyll. (Figure 1b and c). Together, these resultsrevealed species- and cell type-specific Na^+^ accumulation patterns that corresponded to the different salt tolerance strategies of the 2 species (Fig. 1a-c, Figure S1 and S2).

**Figure 1:**
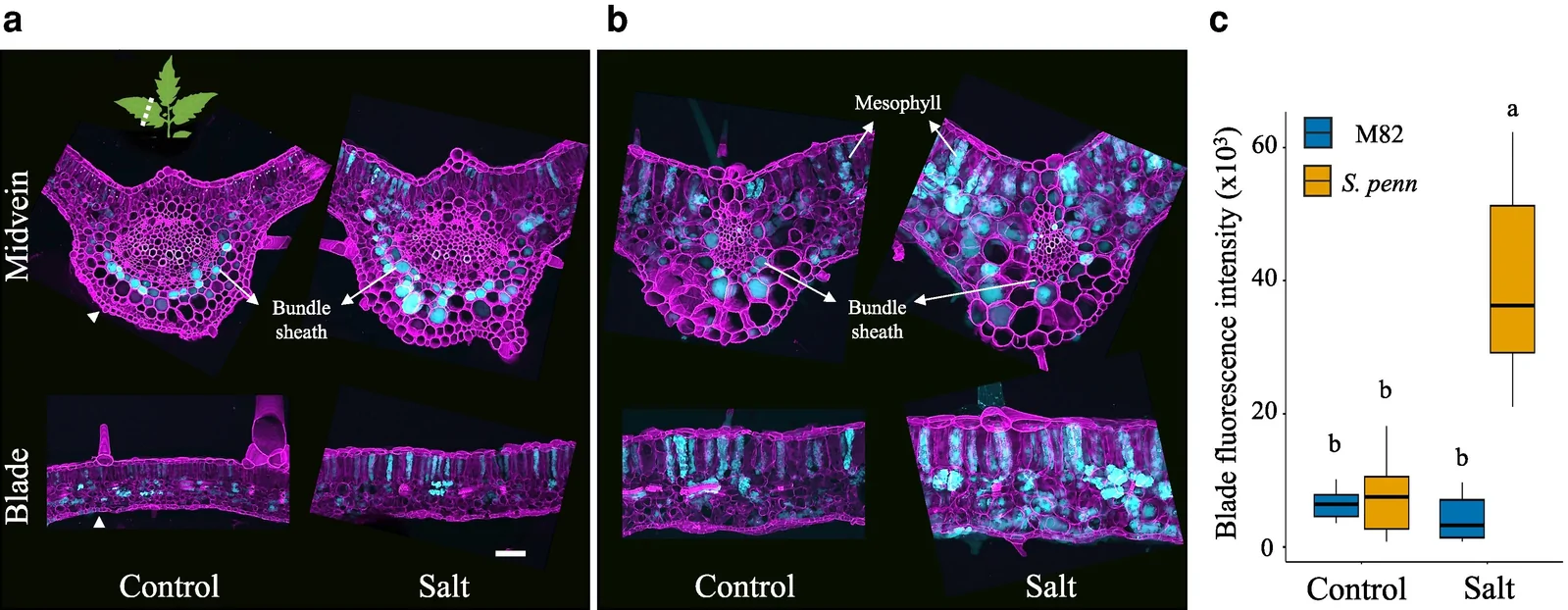
Wild and domesticated tomatoes accumulate Na^+^ in divergent leaf cell domains. Representative live cross-sections of the midvein and leaf blade from the third fully expanded leaf of 5-week-old plants, stained with the Na^+^-specific dye CoroNa Green (cyan) and Congo Red (magenta) to visualize cell walls. Na^+^ preferentially accumulates (a) in M82 in bundle sheath and adjacent parenchyma cells at the abaxial side of the leaf. (b) In *S. pennellii*, Na^+^ accumulation is enriched in the blade mesophyll. Three-week-old plants were exposed to salt stress for 2 wks, starting with 25 mM NaCl and increasing by 25 mM daily until reaching 100 mM NaCl, which was maintained for 9 d. Scale bar indicates 100 µm. (c) Quantification of CoroNa Green signal in blade mesophyll cells (n = 6). Bars are median ± SD. Significant differences (*P* < 0.05) within each condition, detected by 2-way ANOVA followed by Tukey's HSD test, are indicated by different letters.

### Na^+^ accumulation domains are conserved within each species and across other salt-tolerant wild relatives of tomato

Since Na^+^ accumulation is dynamic and occurs preferentially in older than younger leaves (Munns and Tester 2008), we examined whether the observed cellular Na^+^ accumulation patterns are conserved within each species. To this end, we quantified the number of Na^+^-accumulating cells in young and mature leaves within each of 6 anatomical domains (ie, the primary leaflet of the youngest fully expanded leaf and its immediate predecessor, the mesophyll, abaxial parenchyma, bundle sheath, pericycle, vascular bundle, and adaxial parenchyma), after 5 and 9 d of salt stress, as well as in the corresponding control plants. Our analysis revealed that the Na^+^-accumulation domains in the M82 bundle sheath and the *S. pennellii* mesophyll were characteristic of each species and were independent of leaf ontogeny (young or mature leaves), growth conditions (salt stress or control), and severity of salt stress (5 or 9 d of exposure) (Fisher's exact test, *P* < 0.01) (Fig. 2a). Given that *S. lycopersicum* has several wild relatives native to saline environments (Bonarota et al. 2022), we asked if the enriched Na^+^ accumulation in the blade mesophyll is unique to *S. pennellii* or conserved among additional salt-tolerant wild relatives of tomato. To this end, we selected 2 wild relatives of tomato: *S. pimpinellifolium* (LA1589), which belongs to the same clade as *S. lycopersicum*, and *S. peruvianum* (LA1954), which belongs to a separate clade (Fig. 2b). The biomass of each of the 3 wild tomato species, respectively, was not significantly different when comparing control and salt conditions, in contrast to M82, which exhibited a significant biomass reduction in control compared to salt stress conditions (Figure S4a). Unlike M82, *S. pimpinellifolium* and *S. peruvianum* did not show preferential Na^+^ accumulation in the bundle sheath (Figure S4b). Instead, both species exhibited Na^+^ accumulation in the blade mesophyll under salt stress, which was significant in *S. peruvianum* and showed a similar trend in *S. pimpinellifolium* (Figure S2c and d).

**Figure 2:**
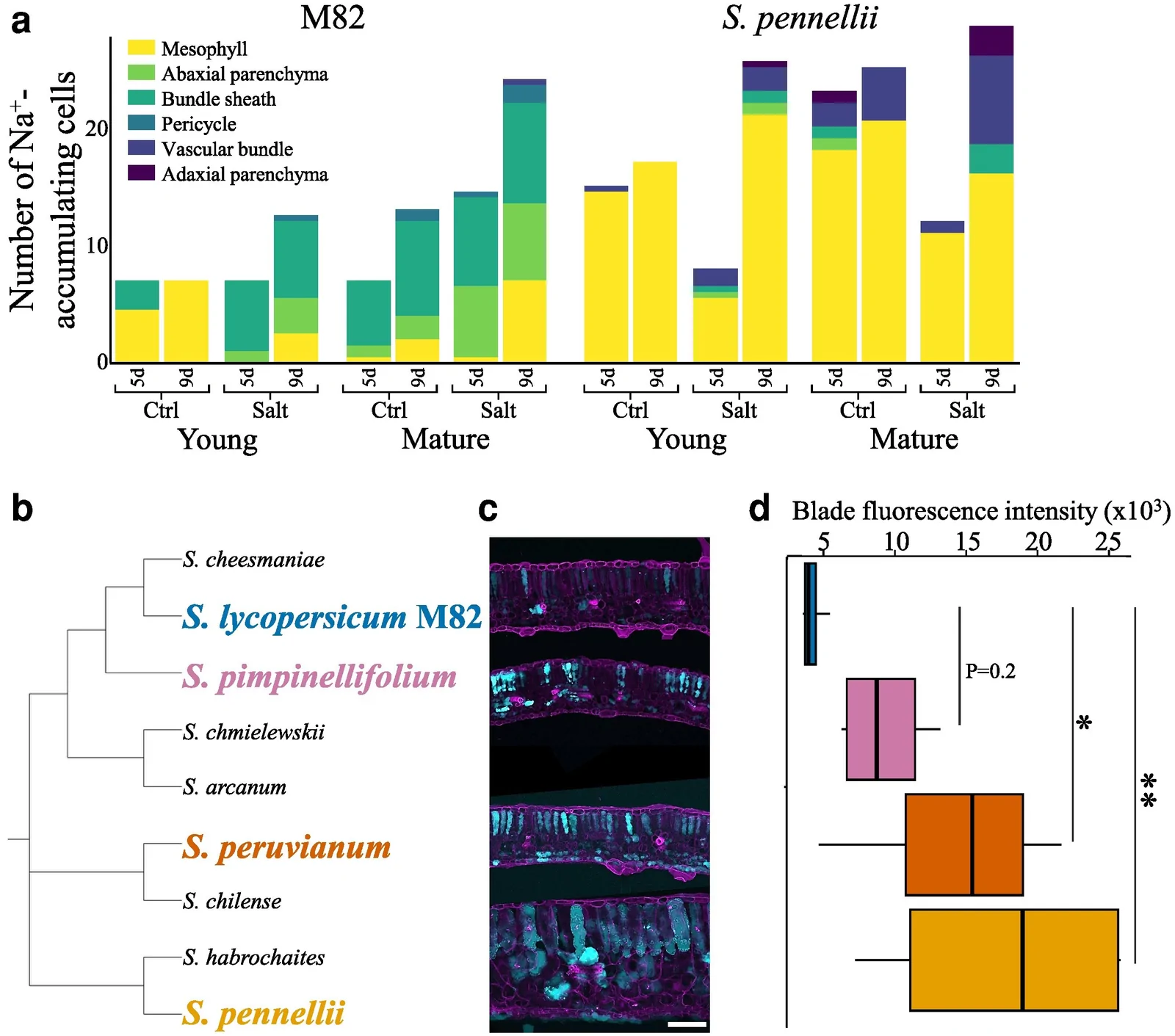
Leaf Na^+^ accumulation domains are conserved both within and across tomato species. (a) Stacked bar plots of the number of cells accumulating Na^+^ among different cell populations in live leaf cross sections, focused on the midveins of young and mature leaves, following 5 and 9 d of salt stress and at the same time points under control conditions (n = 6). Enriched Na^+^ accumulation domains within each species (ie, bundle sheath in M82 and mesophyll in *S. pennellii*) are conserved regardless of the developmental stage of the leaf, growth conditions (salt stress or control conditions), or the duration of the salt stress (Fisher's exact test, *P* < 0.01). (b) Phylogenetic tree based on Grandillo et al. (2011) of domesticated tomato (M82) and selected salt-tolerant wild relatives that are indicated in bold and colored based on the different tree branches. (c) Representative cross sections of live leaf blades from salt-stressed plants. Sections are stained with CoroNa Green (cyan), a Na^+^-specific dye, and Congo red (magenta), which stains the cell wall. The scale bar indicates 100 µm. (d) Quantification of CoroNa Green signal in blade mesophyll cells (n ≥ 4). Significant differences detected by 1-way ANOVA followed by Dunnett's test are denoted with asterisks (** *P* ≤ 0.05, and *** *P* ≤ 0.01).

### Symplastic transport drives the differential Na^+^ accumulation domains

After Na^+^ is delivered to the leaf in the transpiration stream, it initially resides in the xylem apoplast, from where it may either remain within the apoplastic continuum surrounding the veins or be transported across the plasma membrane of xylem parenchyma and bundle sheath cells via Na^+^-permeable transporters and channels, enabling subsequent symplastic redistribution to the blade mesophyll. We hypothesized that the relative contributions of symplastic uptake, apoplastic retention, or apoplastic barriers vary between the 2 species and account for their distinct Na^+^ distribution patterns in the leaf. We therefore traced symplastic and apoplastic pathways and tested for the presence of constitutive or inducible apoplastic barriers around the leaf midveins.

To test the hypothesis that symplastic transport regulates leaf Na^+^ accumulation patterns, we used the fluorescent symplastic tracer 5(6)-Carboxyfluorescein diacetate (CFDA; Jiang et al. 2019) in tomato leaves dark-adapted for 2 h. Dark adaptation substantially reduced photosynthesis, stomatal conductance, and transpiration and attenuated the absolute differences between M82 and *S. pennellii*, thereby minimizing the potential contribution of species-dependent gas-exchange rates to CFDA distribution (Figure S5). We observed a significant decrease in symplastic transport in M82 leaves under salt stress conditions, but not in *S. pennellii* leaves (Fig. 3a, Figure S6a). In the CFDA petiole-feeding assay, this difference was evident from the reduced spread of the fluorescent signal from the veins into surrounding tissues in salt-stressed M82 leaves, whereas *S. pennellii* retained a broad fluorescent distribution. Since reduced symplastic transport is expected to reflect lower plasmodesma permeability, we next used the drop-and-see (DANS) assay (Cui et al. 2015). In this assay, the extent of measured fluorescence spread from the application site reflects cell-to-cell movement and thus plasmodesma permeability. Consistent with the petiole-feeding assay, fluorescent spread was significantly reduced in salt-stressed M82 leaves, but not in *S. pennellii* leaves (Fig. 3b, Figure S6b). Lastly, we examined apoplastic transport using the apoplastic tracer berberine, followed by potassium thiocyanate to remove non-specifically bound tracer and immobilize the remaining signal (Enstone and Peterson 1992). This procedure allows us to qualitatively assess the functional apoplastic pathway in dark-adapted tomato leaves (Figure S5). Apoplastic tracer assays showed that tracer movement in M82 leaves was markedly reduced under salt-stress conditions compared with control conditions. Importantly, however, the dye was not consistently confined to the veins but also accumulated in extravascular regions corresponding spatially to the mesophyll (Fig. 3c), from which Na^+^ accumulation was largely restricted (Fig. 1a and c). In contrast, in *S. pennellii*, the apoplastic tracer accumulated in and around the veins (Fig. 3c), while Na^+^ mainly accumulated in the mesophyll (Fig. 1b and c). Furthermore, staining with basic fuchsin or Auramine O, which binds hydrophobic cell wall components such as lignin and suberin that can accumulate in bundle sheath cells and restrict the apoplastic pathway (Botha et al. 1982), revealed no evidence of an apoplastic barrier around the midveins in either tomato species that can explain the patterns of Na^+^ accumulation (Fig. 3d, Supplementary Figure S6c). Together, these results best support symplastic transport as the predominant driver of leaf Na^+^ patterning under our conditions.

**Figure 3:**
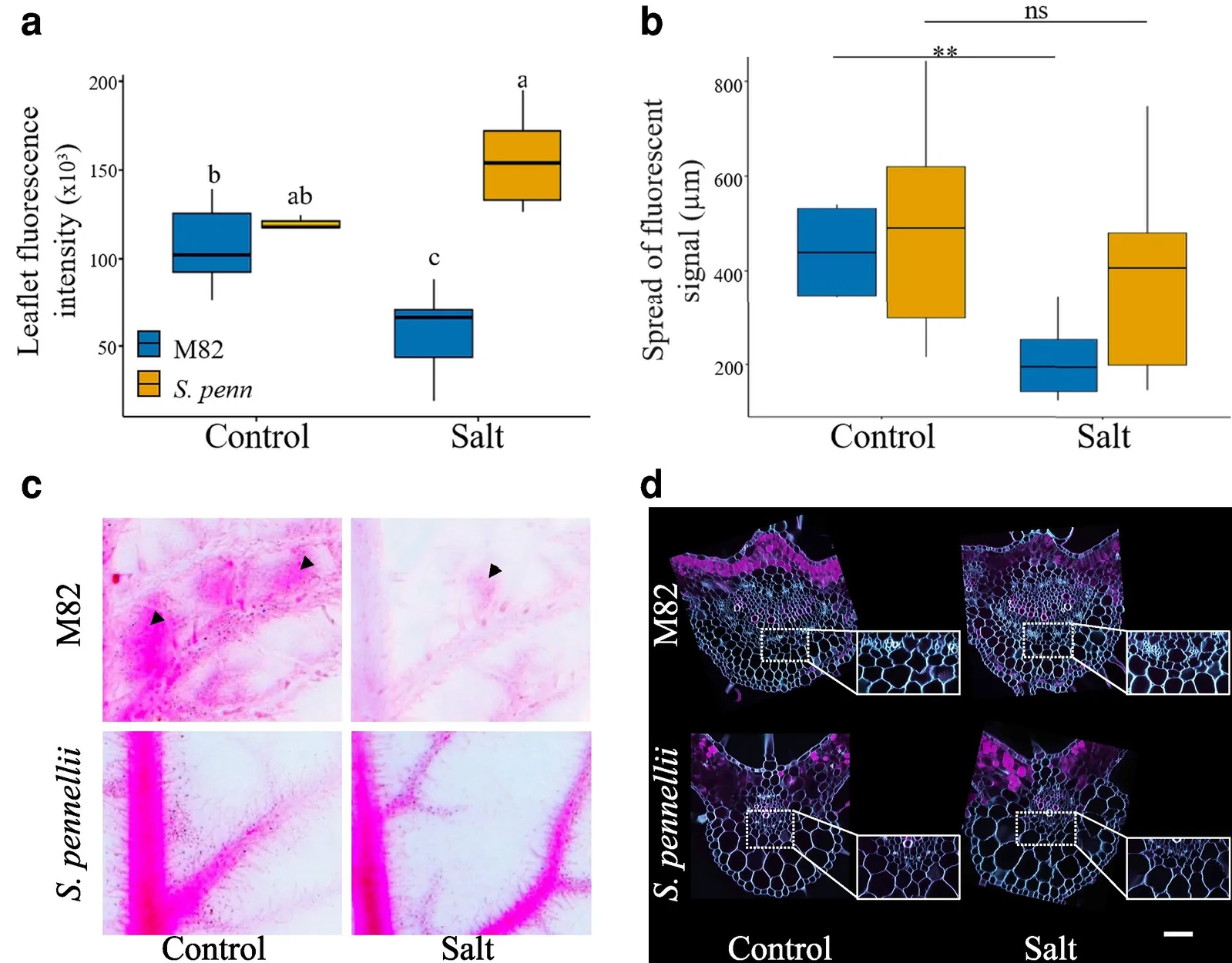
Symplastic, not apoplastic, transport primarily drives the different Na^+^ accumulation domains. (a) Quantification of the symplastic tracer 5(6)-Carboxyfluorescein diacetate (CFDA). Terminal leaflets of the youngest fully expanded leaves were subjected to 2 h of dark acclimation following 2 h of petiole feeding of CFDA (n = 6). Bars are median ± SD. Significant differences (*P* < 0.05), detected by 2-way ANOVA followed by Tukey's HSD test, are indicated by different letters. (b) Quantification of plasmodesma permeability based on CFDA diffusion. Significant differences (*P* < 0.01), detected by Student's *t*-test, are indicated by asterisks; ns indicates a non-significant difference. (c) Representative primary leaflets of the youngest fully expanded leaves were stained with the apoplastic tracer Berberine, followed by chasing with potassium thiocyanate for Berberine immobilization. Dashed lines indicate veins, and arrowheads indicate the tracer accumulation outside the veins. (d) Representative leaf cross sections of the midvein stained for lignin with Basic Fuchsin (magenta) and cellulose with Direct yellow (cyan). Autofluorescence of the chlorophyll is observed in magenta in mesophyll cells. Insets are high-magnification images of the boxed regions, focusing on the bundle sheath and adjacent parenchyma cells that lack consistent or inducible lignin deposition. The scale bar indicates 100 µm.

### A single IL encompasses enriched Na^+^ accumulation domains of both tomato species

To elucidate the genetic basis underlying the species-specific Na^+^ accumulation domains, we utilized an IL population between M82 and *S. pennellii* (LA0716) (Eshed and Zamir 1994, 1995). The population includes 76 homozygous lines, each containing a segment from the wild species (*S. pennellii*) in the background of the domesticated tomato (M82). We first screened for ILs that exhibit phenotypic stability, defined as a non-significant or only marginal biomass reduction under salt stress, similar to *S. pennellii*, while maintaining M82-like biomass under control conditions (Figure S1a). We hypothesized that such phenotypically stable ILs, which perform like *S. pennellii* under salt stress, would also accumulate higher levels of Na^+^ in their leaves (Figure S2a), while retaining M82-like productivity under non-stress conditions. Seven ILs met both criteria (Figure S7) and were selected for quantifying leaf Na^+^ levels using both ICP-MS and CoroNa Green (Figure S8a and c). Based on ICP-MS measurements, among the 7 ILs, only IL6-4 accumulated higher levels of Na^+^ but similar levels of K^+^ compared to M82 under salt stress conditions (Fig. 4a, Figure S8a and b). Additionally, we found that Na^+^ quantification by fluorescence showed a strong positive correlation with ICP-MS measurements (Spearman's ρ = 0.75, *P* = 0.03, Figure S8c), supporting the use of fluorescence intensity as a proxy for relative Na^+^ abundance.

**Figure 4:**
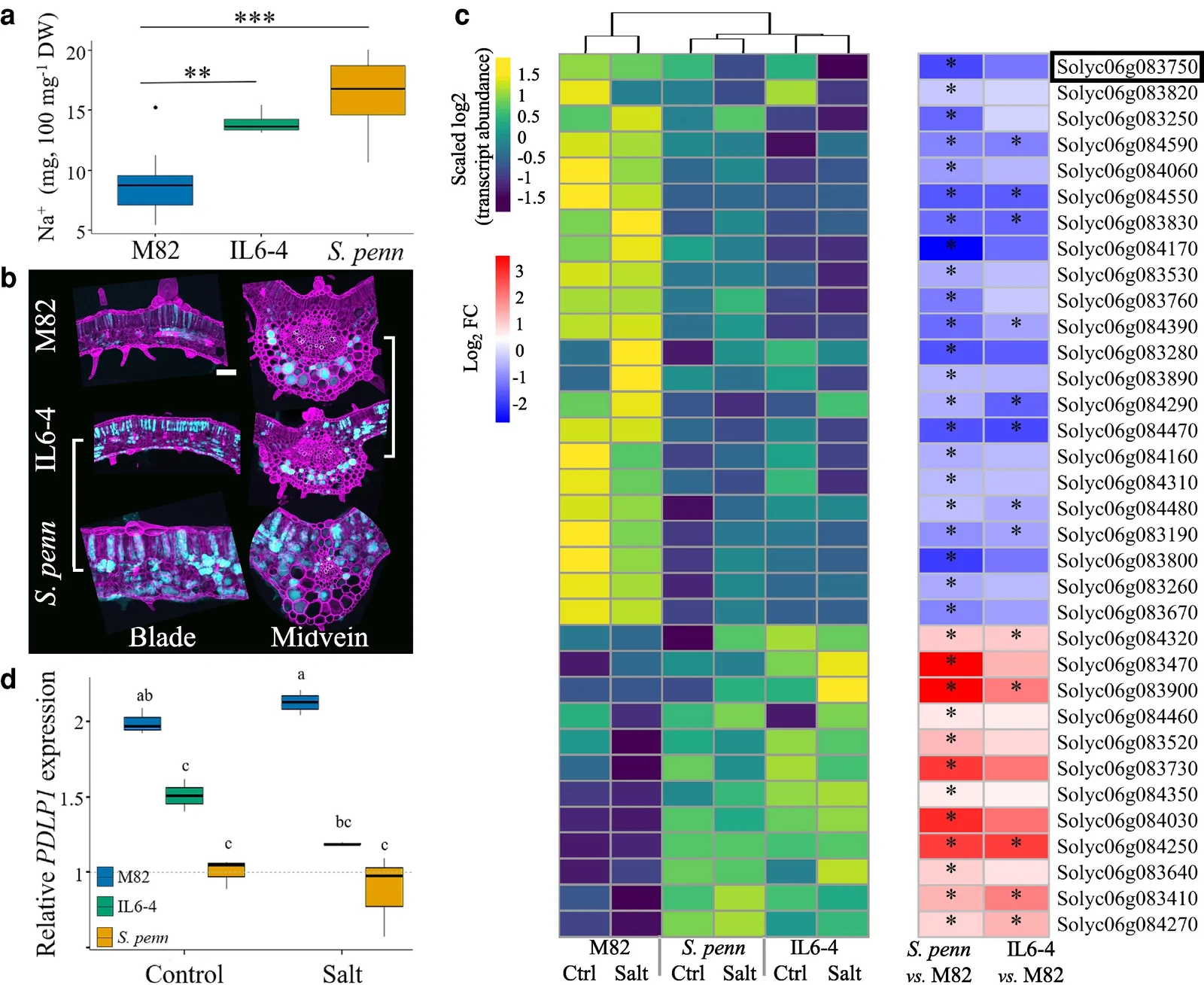
The Na^+^ accumulating introgression line 6-4 (IL6-4) harbors *SpPDLP1,* which shows reduced expression in IL6-4 and *S. pennellii* compared with M82. (a) Box plots of leaf Na^+^ content in plants subjected to salt stress conditions for 12 d (n = 6). Quantification was done with ICP-MS. Significant differences detected by 1-way ANOVA followed by Dunnett's test are denoted with asterisks (** *P* ≤ 0.01, and *** *P* ≤ 0.001). (b) Representative live cross sections of tomato midveins and leaf blades stained with CoroNa Green (cyan), a Na^+^ specific dye, and Congo red (magenta) that stains the cell wall. IL6-4 accumulates Na^+^ at the abaxial side of the midvein, as in the domesticated parent (M82) and in the mesophyll, similar to the wild parents (*S. pennellii*). Scale bar indicates 100 µm. (c) Heatmaps of scaled log_2_ transcript abundance (left) and fold change (FC) values (right) of 34 genes that introgressed from the wild parent into IL6-4 and are *i*) differentially expressed (FDR≤0.1) between *S. pennellii* and M82 under salt stress conditions, and *ii*) maintain similar FC directionality between IL6-4 and M82 under salt stress conditions. Expression was evaluated by bulk RNA sequencing (n ≥ 3). Asterisks indicate differentially expressed genes (FDR≤0.1) between *S. pennellii* or IL6-4 and M82 under salt stress conditions. (d) Box plots showing the relative expression of *Plasmodesmata-located protein 1* (*PDLP1*) orthologs in M82 (*Solyc06g083750*) and *S. pennellii*/IL6-4 (*Sopen06g035160*). Expression was evaluated by RT-qPCR (n ≥ 3), using primary leaflets from the second fully expanded leaf. Exp (SGN-U346908) was used as a housekeeping gene. Bars are median ± SD. Significant differences (*P* < 0.05), detected by 2-way ANOVA followed by Tukey's HSD test, are indicated by different letters.

Tracing and quantification of leaf Na^+^ accumulation in IL6-4 showed that, unlike M82, Na^+^ extended from the abaxial side of the midvein into the leaf mesophyll, similar to *S. pennellii* (Fig. 4b; Figure S8d and e). Because IL6-4 contains enriched Na^+^-accumulation domains derived from both the wild and domesticated parents (adjusted *P* = 0.07; Figure S8e), we hypothesized that introgressed *S. pennellii* gene(s) underlie the elevated Na^+^ levels and their movement into the IL6-4 blade. To test this, we sequenced the transcriptomes of the 3 genotypes under both control and salt-stress conditions (n = 4; Figure S9a–d), focusing on differentially expressed genes (DEGs) within the IL6-4 introgressed region (Chitwood et al. 2013) that display *S. pennellii*-like expression patterns in IL6-4 and differ from M82. Of the 139 introgressed *S. pennellii* genes with M82 orthologs in IL6-4 (Table S2), we identified 34 DEGs (FDR≤0.1) between salt-stressed *S. pennellii* and M82 whose salt-induced fold-change patterns in IL6-4 mirrored those in *S. pennellii* rather than in M82 (Fig. 4c, Table S3). We examined the DEG set for candidates involved in ion transport and compartmentation, particularly via the symplastic pathway. Most DEGs were associated with general metabolism, RNA processing, or protein turnover (Table S3). However, 1 gene, Solyc06g083750, encodes *PLASMODESMATA-LOCATED PROTEIN 1* (*PDLP1*; Figure S9e). In Arabidopsis and aspen, PDLP1 promotes callose deposition at plasmodesmata and thereby reduces plasmodesmata permeability; elevated *PDLP1* levels are generally associated with restricted symplastic transport (Thomas et al. 2008; Tylewicz et al. 2018; Mehra et al. 2022). Consistent with this role, *PDLP1* expression was higher in M82 leaves than in *S. pennellii* and IL6-4, as confirmed by real-time quantitative PCR (RT-qPCR) (Fig. 4d; Table S3). This elevated *PDLP1* expression in M82 is in line with a more restricted symplastic pathway (Fig. 3a and b) and thus supports the confined Na^+^-accumulation domain at the abaxial side of the veins and the lower Na^+^ accumulation in M82 leaves (Fig. 1a and c, Figures S2a, S8d and e). Conversely, the lower *PDLP1* expression in *S. pennellii* and IL6-4 is consistent with enhanced symplastic connectivity in *S. pennellii* (Fig. 3a and b), which could facilitate Na^+^ movement from the bundle sheath into the mesophyll and thereby contribute to the more extended Na^+^-accumulation domains and overall higher leaf Na^+^ levels observed in these genotypes (Figs. 1a and c, 3a and b, Figures S2a, S8d and e).

Additional family members related to *PDLP1* in M82 and *S. pennellii*, as well as their Arabidopsis orthologs, were identified by phylogenetic analysis (Figure S9e). Among the 8 *PDLP* members, 2 additional *PDLPs* (Solyc04g007950 and Solyc09g090680) exhibited induced expression under salt stress conditions in both M82 and IL6-4 compared with *S. pennellii* (FDR≤0.01, Figure S9f, Table S1). These expression patterns identify the 2 genes as candidates for further functional investigation of their potential contribution to the spatial pattern of Na^+^ accumulation.

### *PDLP1* expression is negatively correlated with Na^+^ buildup in the blade mesophyll

Based on the known role of *PDLP1* in establishing symplastic domains (Bayer et al. 2008; Tylewicz et al. 2018; Mehra et al. 2022), and our findings on divergent *PLDP1* expression, symplastic transport, and Na^+^ accumulation patterns between the 2 species, we propose that *PDLP1* is involved in the formation of leaf Na^+^ accumulation domains by regulating symplastic transport. We hypothesize that if increased *PDLP1* expression restricts leaf Na^+^ movement and reduces its accumulation in M82 mesophyll, then wild tomato relatives with higher Na^+^ accumulation in the blade mesophyll should exhibit lower *PDLP1* expression. Using RT-qPCR to analyze *PDLP1* orthologs, we observed lower and similar expression levels among the 3 wild tomato species compared to M82 under salt stress conditions (adjusted *P* ≤ 0.066, Fig. 4d). Furthermore, the expression level of *PDLP1* orthologs was inversely related to Na^+^ accumulation across the 4 tomato species (Spearman's **ρ**=−0.62, *P* = 0.05, Fig. 5b). Collectively, these findings suggest that increased Na^+^ accumulation in the mesophyll of the tested salt-tolerant wild tomatoes is associated with lower *PDLP1* expression, indicating the absence of symplastic domains around the veins in these species.

**Figure 5:**
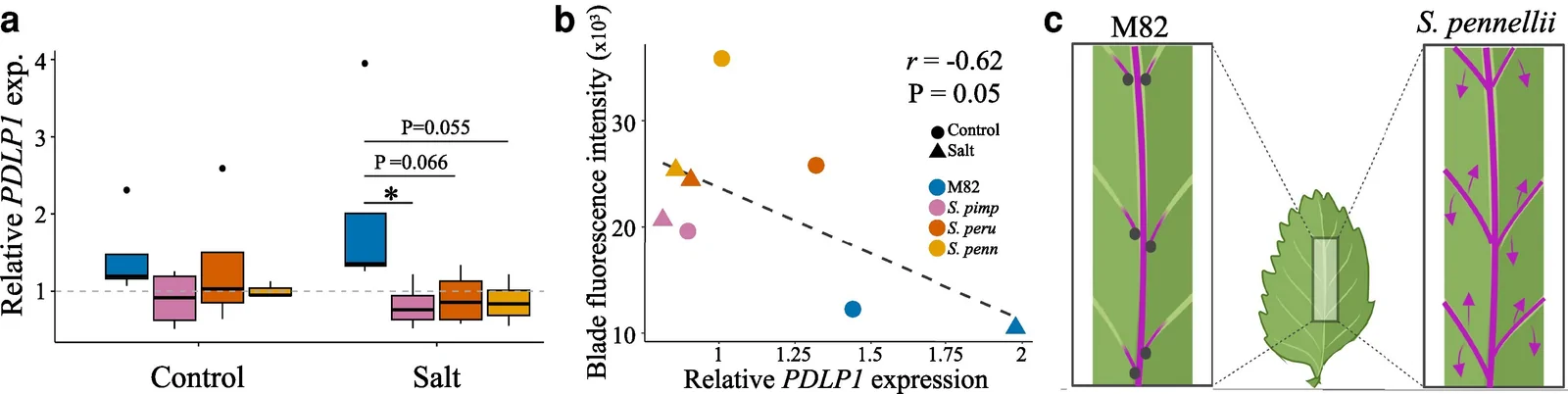
Additional salt-tolerant wild relatives of tomato with increased Na^+^ accumulation in the blade mesophyll also exhibit reduced expression of *PDLP1* orthologs. (a) Box plots of relative expression of *Plasmodesmata-located protein 1* (*PDLP1*) orthologs (n = 4). Expression was evaluated by RT-qPCR (n ≥ 3) using primary leaflets from the second fully expanded leaf. Exp (SGN-U346908) was used as a housekeeping gene. Bars are median ± SD. Significant differences detected by 1-way ANOVA followed by Dunnett's test are denoted with asterisks (* *P* ≤ 0.05). (b) Spearman correlation between mesophyll Na^+^ accumulation (Fig. 2d) and *PDLP1* expression (a) across tomato species grown under control and salt stress conditions. (c) Schematic illustration of the working hypothesis. In M82, the increased and localized expression of *PDLP1* at midvein junctions with second-order veins establishes a symplastic domain that limits cell-to-cell transport of Na^+^ and facilitates its accumulation mainly in bundle sheath and adjacent parenchyma cells. In *S. pennellii*, low *PDLP1* expression enables symplastic transport and Na^+^ accumulation in distal blade mesophyll cells away from the veins.

## Discussion

Despite numerous QTL discoveries and the development of introgressed or transgenic plants, commercially successful salt-tolerant cultivars that include wild tomato alleles remain rare. This may stem from the polygenic and environment-dependent nature of the salt stress response (Bonarota et al. 2022; Guo et al. 2022). Here, we propose that the divergent patterns of leaf Na^+^ accumulation between domesticated and wild tomato species (Figs. 1 and 2) may play a significant role in tissue tolerance and should be considered in breeding efforts to develop salt-tolerant crop varieties.

Several studies have demonstrated cell population-specific expression of Na^+^ transporters, which contribute to the directional retrieval of Na^+^ primarily from xylem vessels via xylem-parenchyma cells. Examples include *HKT1;1* from Arabidopsis, which is expressed in stelar root cells and in leaf xylem-parenchyma, where it mediates Na^+^ unloading from xylem vessels (Sunarpi et al. 2005). Related vascular functions have been reported for rice *HKT1;5* and tomato *HKT1;2,* which are expressed in cells surrounding xylem vessels in roots and shoots and contribute to plant salt tolerance (Jaime-Perez et al. 2017; Kobayashi et al. 2017). Although the previously reported cell type-specific expression of these *HKT* genes confines their activity to vascular-associated cells, Na^+^ retrieval from the xylem affects its subsequent delivery to and accumulation in other cell populations and tissues. Previous studies demonstrated these effects at the organ or tissue level but did not resolve Na^+^ distribution among individual cell populations or directly link cell-specific transporter expression to local Na^+^ accumulation patterns. Such cell-resolved information is needed to define the cellular functions and genetic regulation underlying tissue-level salinity tolerance.

A handful of studies have attempted to assess leaf ion accumulation at the cellular level using various techniques, including cryo-scanning electron microscopy, X-ray microanalysis, and sodium-specific dyes. In salt-treated barley (salt-tolerant cultivar) and wheat (salt-sensitive cultivar), Na^+^ accumulates in leaf tissues but shows no evidence of differential distribution between cell types. This contrasts with other ions, such as Cl^−^, which is preferentially accumulated in epidermal cells, and K^+^, which is more concentrated in mesophyll cells (James et al. 2006). In contrast, in salt-treated *Puccinellia peisonis*, Na^+^ preferentially accumulates in the bundle sheath (Stelzer 1981); in Rhodes grass (*Chloris gayana*), it accumulates in the xylem parenchyma and epidermis (Oi et al. 2022); and in citrus, Na^+^ mainly accumulates in the mesophyll (Gonzalez et al. 2012). These observations suggest that some plants accumulate Na^+^ in distinct leaf cell populations in a species-specific manner, supporting our findings (Figs. 1 and 2). Yet, further research employing standardized methodologies and additional species is needed to identify the cellular and molecular mechanisms underlying enriched Na^+^ accumulation domains in leaves.

After exiting the leaf xylem, Na^+^ can move across the bundle sheath toward the mesophyll and epidermis through apoplastic, transcellular, or symplastic pathways. Previous studies indicate that Na^+^ movement through leaves occurs predominantly through the symplast (Speer and Kaiser 1991; Shapira et al. 2009), consistent with the association between symplastic tracer movement and spatial Na^+^ accumulation observed in our study (Figs. 1 and 3). Because symplastic connectivity is regulated by plasmodesmal permeability, including through callose deposition and the activity of plasmodesmata-located proteins (PDLPs; Thomas et al. 2008; Zavaliev et al. 2011), differences in plasmodesmal regulation provide a potential mechanism underlying the genotype-dependent Na^+^ accumulation patterns observed around the veins. Based on our results and previous reports showing strong spatiotemporal regulation of *PDLP* family members in specific tissues and developmental processes (Thomas et al. 2008; Sager et al. 2020; Li et al. 2024), we hypothesize that the restriction of symplastic transport in M82 may involve localized expression of *PDLP1*, possibly at midvein junctions with second-order veins, which could limit Na^+^ spread into the blade mesophyll and promoting its enrichment on the abaxial side of the midveins (Fig. 5c). The decreased plasmodesma permeability in salt-treated leaves of M82 is accompanied by increased expression of *SlPDLP1*, in contrast to *S. pennellii* and IL6-4, which show lower expression levels of *SpPDLP1* and an extended Na^+^ accumulation domain in the photosynthetically active mesophyll cells of the blade. In accordance with the lower *SpPDLP1* expression and extended Na^+^ accumulation, there is no change in plasmodesma permeability in salt-treated *S. pennellii* leaves (Figs. 1,2&4). Furthermore, our findings demonstrate an inverse relationship between the expression levels of *PDLP1* orthologs in 2 additional salt-tolerant wild relatives of tomato and the enrichment patterns of Na^+^ accumulation in their blade mesophyll (Figs. 2 and 3). Thus, we propose to expand the role of symplastic domains from developmental processes to tolerance strategies in response to salt stress. It is important to note that although our experimental data demonstrate symplastic connectivity as a major contributor to Na^+^ transport, this does not exclude the possibility of some, likely minor, apoplastic movement in the blade mesophyll.

Given the paucity of high-resolution spatial information on Na^+^ accumulation in leaves, this work provides a comprehensive characterization of cellular Na^+^ accumulation patterns in wild and domesticated tomato species, which aligns with their well-documented differences in Na^+^ accumulation at the organ level and their divergent salt tolerance strategies at the whole plant level (ie, Na^+^ includer vs. excluder) (Tal and Shannon 1983). While *PDLP1* expression correlates with foliar Na^+^ patterning, establishing causality will require cell-type-specific perturbations of *PDLP1* and cell-resolved mapping of its expression domain. Upstream processes responsible for whole-plant differences in the observed whole-plant Na^+^ abundance are unknown, but most certainly, the proposed cellular mechanism provides a framework for a better understanding of the regulation of Na^+^ transport and accumulation. This knowledge is necessary to facilitate the precise engineering of tolerance mechanisms to enhance the performance of tomatoes and other vegetable crops under elevated soil salinity.

## Materials and methods

### Plant material

Seeds of *S. lycopersicum* cultivar M82, *S. pennellii* (LA0716), *S. pimpinellifolium* (LA1589), and *S. peruvianum* (LA1954) were obtained from the C.M. Rick Tomato Genetics Resource Center at UC Davis (https://tgrc.ucdavis.edu/). Seeds of the IL population between M82 and *S. pennellii* LA0716 were obtained from the Zamir lab (Eshed and Zamir 1994, 1995).

### Salt stress experiments

Tomato seeds were surface-sterilized with 70% Ethanol for 5 min, followed by 20 min in a fresh 50% bleach solution, and rinsed 5 times with sterile water. Seeds were sown in 12 × 12 cm square Petri plates containing 4.3 g/L MS medium without vitamins (Caisson, MSP09-50LT), 1% (w/v) sucrose, 0.5 g/L MES (pH 5.8) and 1% (w/v) agar (Difco, 214530). Uniform, ∼6-9-day-old seedlings were transferred into 656 mL containers (D40L STUEWE) filled with Profile® Field & Fairway™ (Turface Athletic Profile Field & Fairway clay substrate) and covered with a 2 cm layer of vermiculite. Plants were grown in a Conviron walk-in chamber (22 °C, 70% RH, 16/8 h light/dark cycle, light intensity of ∼150 µmol/m^2^/s) and irrigated every other day with nutrient-rich water (N:P:K, 2:1:2). A completely randomized design, with 6 replicates per treatment and species, was employed starting at 3 to 4 fully expanded leaves (∼3-week-old seedlings). Plants were progressively exposed to salinity by irrigating daily with increasing salt concentrations, starting with 25 mM NaCl and increasing by 25 mM NaCl increments until reaching a target concentration of 100 mM NaCl, which was maintained for 9 d. During this time, plants were watered daily with nutrient-rich water. Altogether, plants were exposed to salt for 14 d. Runoff electrical conductivity was monitored and recorded daily once the target concentration was reached. During the salt treatment, control plants were irrigated daily with nutrient-rich water.

### Screening for salt tolerance introgression lines (ILs)

IL seeds were divided into 5 batches based on their corresponding chromosomes (ie, chromosomes 3, 4, & 8; 2, 5, & 6; 10, 11, & 12; 7 & 9; 1 & low germinating ILs). Each screening assay included the ILs from each batch and the 2 parents: M82 and *S. pennellii* (LA0716). Seeds were surface sterilized with 50% HCl for 15 min, followed by 15 min in a 10% trisodium phosphate solution, then rinsed and sown as described above. Uniform ∼6-9-day-old seedlings from each IL (n = 20), M82 (n = 40), and 32 *S. pennellii* (n = 32) were transplanted into six 72-pot trays filled with profile mix (Turface Athletic Profile Field & Fairway clay substrate) in a completely randomized design and watered with nutrient-rich water. At the stage of 2 fully expanded leaves, half of the plants were exposed to salt stress by irrigating with 50 mM NaCl. After 3 d, the trays were filled with 100 mM NaCl, which was replaced every 2 d. In total, plants were subjected to salt treatment for 12 d. During the salt treatment, control plants were irrigated daily with nutrient-rich water.

### Physiological measurements

On the day of harvesting, Solanum species had 7 to 9 leaves. A fully expanded leaf is defined operationally as a leaf whose terminal and first pair of primary leaflets are completely spread. On harvest day, shoot and washed root samples (n = 6) were oven-dried (60 °C for 4 d) and weighed to determine total plant biomass. The terminal leaflet from the third fully expanded leaf, located below the shoot apex, was collected for measuring relative water content as described in Canto Pastor et al, (2024). Midday measurements were used to estimate osmotic potential at full turgor (Ψs^100^). Primary leaflets from the second fully expanded leaf were placed in vials containing deionized water, rehydrated to full turgor in darkness at 4 °C for 6 h, then blotted dry and snap-frozen in liquid N_2_. Sap osmotic potential was determined with a vapor-pressure osmometer (Vapro 5600, Wescor/METER, United States); osmolality (c_osm_, mOsm kg^−1^) was converted to osmotic potential at full turgor as Ψ_s_^100^ (MPa) = −0.00248 × c_osm_ (25 °C). OA was calculated as OA = Ψ_s_^100^ control - Ψ_s_^100^ stress, so that positive values indicate greater solute accumulation. Photosynthesis rate (A), stomatal conductance (gs), and transpiration rate were measured between 9:30 and 11:30 AM under light conditions and following 2 h of dark adaptation on the abaxial surface of the terminal leaflet from the third or fourth fully expanded leaf using a LICOR-6400XT portable photosynthesis system (LI-COR Biosciences, Lincoln, Nebraska, United States). Light intensity was set to a high, near-saturating level of 1,000 μmol m^−2^ s^−1^, with a constant air flow rate of 400 μmol s^−1^ and a reference CO_2_ concentration of 400 μmol CO_2_ mol^−1^ air.

### ICP-MS elemental analysis

On the day of harvesting, leaves and washed root samples of M82 and *S. pennellii* were oven-dried (60 °C for 96 h), homogenized, and measured for their dry weight. Samples were digested in concentrated nitric acid and analyzed by inductively coupled plasma mass spectrometry (ICP-MS) at the UC Davis/Interdisciplinary Center for Plasma Mass Spectrometry (UCD/ICPMS) to measure Na and K content within each tissue. Leaf samples of M82, *S. pennellii,* and 7 selected ILs from the salt tolerance screening assays were prepared the same way as for UCD/ICPMS and measured at the UC Berkeley College of Natural Resources ICP Spectroscopy Facility. Each genotype, tissue, and condition had at least 6 biological replicates.

### Histochemistry and imaging

#### Sodium staining and viability staining

Relative Na^+^ enrichment within foliar cell populations was determined following 12 or 14 d of salt treatment, using live leaflet cross-sections from the third fully expanded leaf within 24 to 32 h of sampling. To assess the conservation of Na^+^ enrichment within distinct cell populations between M82 and *S. pennellii*, primary leaflet samples were collected from the first fully expanded leaf (ie, young leaf) and third to fifth fully expanded leaves (ie, mature leaves), following 5, 9, and 14 d of exposing plants to salty water. In parallel, leaflets from control plants were sampled at the corresponding developmental stages.

One-centimeter cuts from the middle third of the leaflet spanning the midvein were embedded in 4% (w/v) agar plugs (n = 6). Plugs were kept in scintillation vials containing fresh incubation buffer (20 mM MOPS [Thomas Scientific, C791M00], pH 8 (adjusted with KOH), 0.5 mM calcium sulfate [Spectrum, CA171], and 200 mM sorbitol [Amresco, 0691]). Within 2 h of sampling, 300 µm sections were prepared using a vibratome (Leica, VT1200S) and stored in a 12-well plate containing incubation buffer. Fifty micrograms of CoroNa Green were first dissolved in 100 μl dimethyl sulfoxide to prepare the stock solution. Four sections (ie, technical replicates) from each genotype and treatment were transferred to a new well and incubated for ∼18 h in the dark at room temperature, with gentle shaking, in 250 µL of 0.1 mM CoroNa Green reagent (ThermoFisher, C36676) diluted in incubation buffer. In parallel, 3 sections from each genotype and treatment were transferred to a new well containing incubation buffer alone and served as unstained controls for autofluorescence and viability tests. Before imaging Na^+^ accumulation or control sections to assess background autofluorescence, freshly prepared 0.15% (w/v) Congo red (MP, 105099), reconstituted in incubation buffer, was added to a slide containing the section and incubated for ∼1 min. Congo red, which stains cellulose, was used to mark the different cell layers of the cross-section (Mitra and Dominique 2014).

Samples were imaged with a Zeiss LSM 900 confocal microscope (water immersion, ×20 objective), with excitation at 488 nm and emission collection of 493–524 nm for CoroNa Green and 591–650 nm for Congo red. Z-series sections were imaged at 4.5-μm intervals.

Because cellular sodium sequestration requires live cells, we validated cell viability of our sections using fluorescein diacetate (FDA; Sigma-Aldrich, F7378), whose fluorescence depends on cell membrane integrity (Jones et al., 2016). A fresh stock solution of 0.2% (w/v) FDA in acetone was prepared, diluted to 4 μg/mL in incubation buffer, and kept in the dark. Since the FDA signal quickly fades, samples were incubated for ∼30 min in the dark at room temperature in a fresh working solution of FDA and then imaged using a Zeiss LSM 700 confocal microscope (water immersion, ×20 objective), with excitation at 488 nm and emission detected using the eGFP filter at 500–550 nm. Z-series sections were imaged at 4.5-μm intervals. Autofluorescence signals from unstained samples were collected using identical imaging settings as those used for stained samples.

#### Leaf cellular anatomy characterization

Characterization of tissue anatomy and identification of the Na^+^-accumulating cell types on the abaxial side of the midvein were performed on M82 leaflets from the third fully expanded leaf (n = 6) using Toluidine Blue O (TBO; Baker, W143–03) and an iodine-potassium iodide (IKI) solution. Sections, either 300 or 150 µm for IKI and TBO, respectively, were fixed overnight with Formalin-Aceto-Alcohol (FAA) solution (v/v; 50% ethanol 95%, 5% glacial acetic acid, 10% formalin, 35% water) followed by gradual rehydration by 30 min incubations in 70%, 50%, 30% and 10% (v/v) ethanol. Fully rehydrated samples were stored in water at 4 °C. To delineate leaf anatomy and visualize tomato's bicollateral veins, we used 0.1% TBO (w/v), a metachromatic dye that is used to differentiate between primary and secondary cell walls and assist in orienting the position of the xylem, phloem, and surrounding cell layers (Yeung 1998). Amyloplast-accumulating bundle sheath cells were detected using the starch-specific dye IKI, following the protocol specified in Yeung (1998). Sections were imaged using a bright-field Olympus AH-2 microscope (water immersion, ×20 objective).

Fixed sections (FAA, 150 µm) of M82 and *S. pennellii* primary leaflets from the third and fourth fully expanded leaves (n = 6) were used to determine if cell walls of the bundle sheath and adjacent parenchyma contain hydrophobic compounds. To this end, we used basic fuchsin (Sigma, 857343-25G), together with Direct Yellow 96 (Biosynth, FD41192), which stains lignin and cellulose, respectively, and Auramine O (Sigma-Aldrich, 861030), a lipophilic fluorescent dye that stains suberin and other hydrophobic compounds, such as cutin. Following Ursache et al. (2018), for lignin and cellulose staining, sections were stained in Clearsee solution with 0.2% (w/v) basic fuchsin for 30 min, washed once with Clearsee solution, and then stained with 0.1% (w/v) Direct Yellow 96 (in Clearsee) for 1 h. Sections were then washed twice with Clearsee solution and imaged with a Zeiss LSM700 confocal microscope (water-immersion ×20 objective). Basic fuchsin was imaged with 555 nm excitation, and emission collection of 600–650 nm. Direct Yellow 96 was imaged with 488 nm excitation, and emission collection of 500–550 nm. For suberin staining, sections were incubated in Clearsee solution containing 0.5% (w/v) Auramine O overnight, then washed twice for 2 h in Clearsee solution and finally imaged using a Zeiss LSM700 confocal microscope (water-immersion ×20 objective). Auramine O was imaged with 488 nm excitation, and emission collection of 505–530 nm. Autofluorescence was imaged with 555 nm excitation and emission collected between 560 and 800 nm.

#### Transport assays and plasmodesmal permeability

Tracing of leaf apoplastic and symplastic transport pathways was done using primary leaflets from the second fully expanded leaf. Apoplastic tracing is based on Enstone and Peterson (1992), with the following modifications: Following 14 d of salt exposure, M82 and *S. pennellii* plants (n = 6) were transferred to a dark room for 2 h to minimize differences in transpiration rates resulting from growth conditions. Following dark acclimation and while kept in the dark, primary leaflets (∼2 cm long) were sampled under a green light, and their petiolets were immediately immersed in pre-labeled single PCR tubes containing either freshly prepared apoplast tracing solution of 0.05% (w/v) Berberine Hemisulfate (Sigma-Aldrich, B3412-10g) in water, or one of the two control solutions: 90 mM potassium thiocyanate (KSCN; Sigma-Aldrich, 207799-100G), dissolved in water or water alone. Samples were incubated in the dark for 2 h, ensuring only the petiolets were completely immersed in the solution throughout the incubation period. Samples incubated with Berberine Hemisulfate were then transferred to new PCR tubes containing KSCN for 2 h to induce precipitation of berberine thiocyanate within the tissues. Finally, images of the abaxial side of the leaflets were acquired on a Zeiss Discovery V12 fluorescence stereoscope equipped with an AxioCam MR3 camera and an X-Cite 120 illuminator, using a GFP filter set (Ex 470/40, DM 495 LP, Em 525/50).

The tracing of symplastic transport is based on Jiang et al. (2019), with modifications described above for the plants’ dark acclimation. Leaflets were sampled (n = 6), and their petiolets were immersed in pre-labeled single PCR tubes containing either freshly prepared symplastic tracing solution of 5 μM 5-(and-6)-Carboxyfluorescein Diacetate (CFDA; Sigma-Aldrich, C195) or water. Samples were incubated in the dark for 2.5 h and imaged as described above.

Plasmodesmal permeability was independently quantified following the DANS assay (Cui et al. 2015) on the primary leaflet (n = 6) from the fourth fully expanded leaf. The standard protocol was modified by supplementing both the CFDA working solution and the whole-leaf imaging medium with 0.015% (v/v) Tween-20 to improve contact with the leaf surface.

#### Image analysis

Because CoroNa Green is an intensity-based dye that predominantly reports vacuolar Na^+^ in intact plant cells, measurements were interpreted as relative enrichments within specific cell populations rather than as absolute cytosolic concentrations. The intensity of the CoroNa Green signal was quantified using ImageJ version 1.54 g. All confocal Z-stack images were projected using maximal intensity projection. Next, for each image, we split the image channels and analyzed only the green channel for area, mean, and integrated Density. Using the freehand tool, the region of interest (ROI) within the leaf cross-section, as well as the background regions of the image (outside the cross-section), was selected. Five to six technical measurements were obtained for the ROI and background regions in each image. Technical measurements were averaged, and the intensity of CoroNa Green signal was calculated based on corrected total cell fluorescence using the following equation: Integrated density of ROI − (Area of ROI × Mean background fluorescence); all images were processed with identical thresholds. To estimate the number of Na^+^ accumulating cells among leaf cell populations, we first defined the cell population of interest within and around the midvein: adaxial parenchyma, abaxial parenchyma, mesophyll, bundle sheath, pericycle, and inner vascular bundle cells. For each maximal intensity projection image, CoroNa Green-positive pixels were identified in Fiji/ImageJ using Adjust → Color Threshold with the following fixed settings (Color space = HSB, Threshold color = B&W, Method = Default, Dark background = on). Brightness thresholds were set to 55–255, while Hue and Saturation were kept at full range, generating a binary mask of positive signal. The number of Na^+^-accumulating cells in each anatomical category was then recorded. Because cell counts across anatomical categories varied among samples and species, the data are presented as counts rather than percentages. All images were processed with identical parameters. Quantification of CFDA signal intensity was performed as described in Cui et al. (2015). Some images were adjusted for brightness and contrast using ImageJ and assembled into figures in PowerPoint.

#### RNA-Sequencing and data analysis

M82, *S. pennellii*, and IL6-4 plants were grown as described above. Following 13 d of irrigation with salt water, primary leaflets from the second fully expanded leaf were sampled and immediately flash-frozen in liquid nitrogen. RNA was extracted from 4 leaf samples (ie, biological replicates) per genotype and condition using the Direct-zol RNA MiniPrep Plus kit (Neta, RPI-ZR2053). Sequencing libraries were generated as previously described in Manzano et al. (2025). RNA-seq libraries were pooled and sequenced using the Illumina NovaSeq (PE150). Since the Lexogen QuantSeq FWD 3′ mRNA-Seq kit was used to prepare the libraries, only Read 1 (R1) reads were kept for downstream analysis. Sequences were pooled and trimmed using Trim Galore (v.0.6.6). Genome indices were built with STAR (Pebesma and Bivand 2023) for *S. lycopersicum* SL4.0 (ITAG4.0) (Tomato Genome 2012) and *S. pennellii* v2.0 (Bolger et al. 2014) (--sjdbGTFtagExonParentTranscript Parent). Trimmed reads were aligned with STAR, and gene counts were generated with HTSeq-count against the respective GFF3 annotations (--type exon, --idattr Parent). To select a reference genome, we compared mapping statistics (eg, uniquely mapped reads, multimapping, and mismatch rate), annotation completeness/quality, and sample clustering using principal component analysis (PCA) across the 3 genotypes and conditions. Because mapping metrics were largely similar, PCA structures were nearly indistinguishable between references (Figure S5a-d), and the *S. lycope*rsicum annotation is more complete and better curated, we used the *S. lycopersicum*-based alignments for all downstream differential expression analyses. DEGs were identified using the limma R package as described in Manzano et al. (2025). Genes with an adjusted *P-value* (P_adj_) ≤ 0.1 were considered differentially expressed (Table S1). The MapMan annotation file, based on ITAG4.0 (https://mapman.gabipd.org/mapmanstore), was used for functional gene annotations. Since we focused on DEGs within the introgressed region of IL6-4, we used the following approach to identify them: we intersected the 146 *S. pennellii* genes harboring in bin d-6G (IL6-4), based on Spenn-v2-cds-annot.fa file (https://solgenomics.net/ftp/genomes/Solanum_pennellii/), with the 162 *S. lycopersicum* genes that were introgressed out of d-6G, based on Chitwood 2013 and following update to ITAG3.2. The orthology relationship between the 2 species was determined using an ortholog pair file kindly provided by Prof. Julin Maloof (UCD). BLASTn (https://blast.ncbi.nlm.nih.gov/Blast.cgi) was used to further identify *S. lycopersicum* orthologs among 21 *S. pennellii* genes that were absent from the ortholog pair file. Altogether, *S. lycopersicum* orthologs were assigned to 139 *S. pennellii* genes (Table S2).

#### RNA extraction and real-time (RT) PCR

To validate the expression of *PDLP1* (Solyc06g083750) in M82 and its orthologs in IL6-4 and tomato wild relatives (*S. pennellii*, *S. pimpinellifolium*, and *S. peruvianum*), plants were grown, and RNA was extracted from primary leaflets from the second fully expanded leaf as described above for the RNA-seq experiment with 3-4 biological replicates per genotype and treatment. First-strand cDNA was synthesized using SuperScript^TM^ III reverse transcriptase (ThermoFisher, 18080-044) following the manufacturer's instructions. RT-qPCR was performed using PowerUp™ SYBR™ Green Master Mix (ThermoFisher, A25742) on the CFX96TM Real-Time PCR Detection System (Bio-Rad Laboratories Inc.). *PDL­P1*-specific primers were designed using the *S. lycopersicum* and *S. pennellii* genomes in Primer-BLAST software (Ye et al. 2012) (Table S4). The SnapGene software (https://www.snapgene.com/) was used to align the CDS sequences of *PDLP1* orthologs from the 4 tomato species, ensuring that the amplicon sequences were identical. The 2^−ΔΔCT^ method (Livak and Schmittgen 2001) was used to normalize and calibrate transcript values relative to the housekeeping gene Expressed (SGN-U346908) (Exposito-Rodriguez et al. 2008).

#### Phylogenetic analysis

The phylogenetic tree was generated as described by Kajala et al. (2021). Briefly, homologous sequences were identified by BLASTP (Boratyn et al. 2013), aligned with MAFFT v7 (Katoh and Standley 2013), trimmed with trimAl (Capella-Gutierrez et al. 2009), and used to construct a maximum-likelihood tree in RAxML (PROTGAMMAAUTO, 100 bootstrap replicates); poorly supported branches were then collapsed.

#### Statistical analysis and reproducibility

Statistical analyses were conducted in the R environment (v. 4.3.3), and derived plots were created using ggplot2 (v. 3.5). For multiple comparisons between genotypes, a 2-way or 1-way ANOVA was performed, followed by a Tukey-Kramer post hoc test or Dunnett's test when compared with M82, respectively. Enrichment of Na^+^ accumulation within a specific cell population was determined based on Fisher's exact test. Correlation between Na^+^ quantification using ICP-MS or *PDLP1* expression and fluorescent intensity of the CoroNa Green signal, respectively, was done using the Spearman method. Statistical analysis of phenotypic stability within the IL population was done using Student *t*-tests in Microsoft Excel v.1808. Individual *P* values for differences of selected ILs between M82 and S. pennellii under control and salt stress conditions, respectively, are provided in Figure S3. For all box plots, the center depicts the median, and the lower and upper box limits depict the 25th and 75th percentiles, respectively. Whiskers represent minima and maxima. Closed dots depict individual samples. In all cases, the number of individual biological samples is denoted as n. Experiments and representative images involving histological staining were repeated at least twice independently, except for the apoplastic transport assay.

## Supplementary Material

kiag779_Supplementary_Data

## Data Availability

Raw sequencing files of mRNA sequencing are available at the Short Read Archive of the National Center for Biotechnology Information (https://trace.ncbi.nlm.nih.gov/Traces/sra) under accession number GSE338566.
